# ABR-UNet3D: Aspect-Aware Boundary-Resilient Attention for Robust Cardiac MRI Segmentation

**DOI:** 10.3390/diagnostics16111598

**Published:** 2026-05-23

**Authors:** Serdar Akyel, Zeki Cetinkaya, Fatih Topaloglu, Eser Sert

**Affiliations:** 1 Afyonkarahisar State Hospital, Ministry of Health, 03030 Afyonkarahisar, Türkiye; drserdarakyel@hotmail.com; 2 Elazığ Fethi Sekin City Hospital, Ministry of Health, 23280 Elazığ, Türkiye; zeki2387@gmail.com; 3 Department of Computer Engineering, Faculty of Engineering and Natural Sciences, Malatya Turgut Ozal University, 44210 Malatya, Türkiye; fatih.topaloglu@ozal.edu.tr

**Keywords:** cardiac magnetic resonance imaging, 3D semantic segmentation, UNet3D, attention mechanisms, Aspect-Aware Complementary Attention, deep learning

## Abstract

**Background:** Cardiac magnetic resonance (MRI) images often exhibit low contrast, anatomical variability, and indistinct boundaries, particularly in the myocardium (MYO) and right ventricle (RV). These challenges can reduce the reliability of both manual and automated segmentation, highlighting the need for more robust and boundary-aware approaches. **Methods:** In this study, an Aspect-Aware Boundary-Resilient UNet3D (ABR-UNet3D) architecture is proposed for cardiac MRI segmentation. The model incorporates an Aspect-Aware Complementary Attention (AAC) module that combines multi-planar contextual information with a complementary gating mechanism to enhance boundary representation. The method was evaluated on the ACDC dataset under consistent training conditions. In addition to Dice Similarity Coefficient (DSC) and Intersection over Union (IoU), boundary-based metrics, including the 95th percentile Hausdorff Distance (HD95), Average Surface Distance (ASD), and Surface Dice, were employed. Furthermore, a five-fold cross-validation protocol and detailed ablation studies were conducted to assess robustness and analyze the contribution of individual AAC components. **Results:** The proposed method achieved a mean DSC of 0.9603 in single-run experiments on the ACDC dataset and showed consistent performance in anatomically challenging regions, particularly for RV and MYO segmentation. In addition, five-fold cross-validation experiments resulted in an average DSC of 0.952 ± 0.009 and IoU of 0.908 ± 0.012, indicating stable performance across different data splits within the evaluated dataset. Boundary-based metrics also showed improved surface agreement and lower boundary errors compared with the evaluated baseline models. Ablation studies further indicated that the combined use of multi-planar contextual information and complementary gating contributes more effectively to segmentation performance than the individual components used separately. **Conclusions:** The results suggest that the proposed ABR-UNet3D architecture provides a stable and competitive segmentation framework for cardiac MRI images within the scope of the ACDC dataset. By jointly modeling contextual information and boundary refinement, the method improves segmentation reliability in challenging regions while maintaining competitive and consistent performance with respect to existing approaches.

## 1. Introduction

Heterogeneity in the myocardial tissue, edema-like changes in appearance, and decreased contrast between adjacent tissues can all be caused by certain pathological or physiological situations in cardiac MRI. The myocardium (MYO) and right ventricle (RV) boundaries are very ambiguous in this scenario, which makes segmentation challenging for both automated and human methods. The reliable computation of parameters frequently used in the quantitative assessment of cardiac function, such as ejection fraction, ventricular volumes, and myocardial mass, depends critically on the accurate and consistent segmentation of the right ventricle (RV), myocardium (MYO), and left ventricle (LV).

In the area of medical image segmentation, deep learning (DL)-based techniques have recently offered notable benefits over traditional methods. Convolutional neural network (CNN)-based encoder–decoder architectures are particularly popular because of their effectiveness at modeling multi-scale contextual information. Because it can immediately learn three-dimensional spatial context from volumetric data, UNet3D stands out among these designs as a powerful foundational technique in CMR segmentation [1]. Compared to two-dimensional methods, UNet3D can more accurately model inter-slice continuity and enhance anatomical consistency.

Though it provides a solid foundation, the typical UNet3D design has many drawbacks when it comes to accurately depicting intricate heart anatomy. Especially in low-contrast regions and in MYO and RV structures with high boundary ambiguity, over-reliance on local features can lead to erroneous segmentations. To overcome this problem, attention mechanisms have become widely used in the literature. Channel attention (e.g., squeeze-and-excitation blocks [2]), spatial attention, and hybrid methods combining both approaches aim to improve performance by enabling the network to focus on relevant features. However, a significant portion of current attention-based approaches either focus on only a single spatial perspective or implement the attention mechanism in a limited number of layers. Yet, cardiac structures exhibit different geometric and anatomical characteristics along the axial, sagittal, and coronal planes. This multi-planar nature cannot be adequately represented by single-axis or single-type attention mechanisms. Although transformer-based or hybrid architectures in the literature partially address this problem [3,4], these approaches are often limited in clinical applications due to high computational costs and memory requirements. On the other hand, while many studies in the literature focus on architectural innovations, they do not address the training and evaluation process from a holistic perspective. Especially in medical image segmentation, not only volumetric overlap metrics but also surface-based metrics that measure boundary accuracy are of great clinical importance [5].

In this study, a segmentation architecture called Aspect-Aware Boundary-Resilient UNet3D (ABR-UNet3D) is proposed, enhanced with an Aspect-Aware Complementary Attention (AAC) module integrated into the UNet3D architecture to overcome the limitations. The proposed AAC structure generates channel-attention vectors by combining global feature descriptors obtained from three different anatomical planes (XY, XZ, and YZ) and additionally enhances feature maps with a complementary spatial gate mechanism. In this way, the network can simultaneously learn both multi-plane global contextual information and local spatial details. Therefore, commonly used attention modules such as SE or CBAM may not be sufficient to model plane-specific contextual cues in 3D volumes, especially in regions with high boundary ambiguity. To address this limitation, the proposed AAC module was developed.

The proposed ABR-UNet3D approach has been extensively evaluated through experimental studies conducted on the ACDC dataset [6]. The training, validation, and testing processes were addressed end-to-end; Dice and IoU metrics were reported both on average and at the class level. According to experimental data, the proposed AAC integration showed consistent performance improvements on the ACDC dataset, notably in the RV and MYO classes where segmentation is very difficult and border ambiguity is considerable.

The main contributions of this study can be summarized as follows:

- To enhance segmentation performance in cardiac MRI images with low contrast and fuzzy boundaries, a complementary gating mechanism is devised. In cardiac MRI images, myocardial tissue usually has uneven boundaries and weak contrasts. By improving feature representations in these ambiguous areas and boosting boundary sensitivity via a 1 × 1 × 1 convolution-based complementary gate, the proposed Aspect-Aware Complementary Attention (AAC) structure expands on traditional channel-attention techniques.
- The reconstruction of anatomically complex and poorly defined cardiac structures is improved by integrating the AAC module at the decoder stage. The AAC module produces more consistent segmentation results by carefully reweighting multi-scale features transferred from the encoder, especially in difficult areas like the myocardium, right ventricle (RV), and left ventricle (LV).
- The proposed framework was evaluated on the ACDC dataset under a controlled experimental protocol. When tested on the ACDC dataset, the ABR-UNet3D model demonstrated strong performance in difficult scenarios involving border ambiguity, structural heterogeneity, and low contrast. The stability and robustness of the proposed framework were further validated using a five-fold cross-validation protocol.

The remainder of this paper is organized as follows. Section 2 reviews related work on cardiac MRI segmentation. Section 3 introduces the proposed ABR-UNet3D architecture and its Aspect-Aware Boundary-Resilient Attention module. Section 4 presents the experimental setup and segmentation results. Section 5 discusses the findings and limitations of the study, and Section 6 concludes the paper.

## 2. Literature Review

Because of their anatomical variety and structural complexity, automated 3D segmentation of the heart and circulatory structures is still difficult. The literature is increasingly emphasizing machine learning (ML) and deep learning (DL) techniques based on artificial intelligence (AI) to address these challenges. For this objective, a number of CNN-based techniques have been put forth [7,8]. Due to significant gains in cardiac structure segmentation brought about by recent developments in deep learning, fully convolutional networks (FCNs) [9,10], U-Net designs [11,12,13], and UNet3D-based models have become widely used. Furthermore, more efficient representation of spatial and contextual information in 3D medical images has been made possible by single-view [14,15,16], multi-view [17,18], and attention-based techniques [19,20,21,22]. Multitask learning frameworks and hybrid models that combine CNN-based techniques with complementary approaches have also been investigated to further maintain anatomical consistency [23,24,25].

### 2.1. Convolutional Neural Network-Based Medical Image Segmentation

Convolutional neural network (CNN)-based techniques have demonstrated excellent performance in a variety of two-dimensional (2D) and three-dimensional (3D) medical picture segmentation applications since the launch of the U-Net architecture [26]. As a result, U-Net-based networks have been widely adopted in the literature [26,27,28,29,30,31]. In this context, many enhanced U-Net variants have been proposed, including IRA-UNet [32], Attention-UNet [33], ICA-UNet [34], ResUNet [35], and UNet++ [36]. In particular, Attention-UNet increases the encoder’s focus on task-relevant features by incorporating attention gates into the skip connections between the encoder and decoder. Architectures such as DS-UNeXt [37] and N-Net [38] have also been introduced as improved models tailored for cardiac image segmentation.

Zhou et al. [39] proposed U-Net++, which introduces dense skip connections across multiple decoder levels to improve feature reuse and information flow. Before combining encoder and decoder features, Liu et al. [40] improved feature representation by adding a residual path (ResPath) with extra convolutional processes. Volumetric networks analyze 3D volumes made up of consecutive slices or multimodal inputs directly, allowing them to better utilize spatial context in 3D segmentation. However, to balance segmentation accuracy and computing efficiency, Isensee et al. [41] used multi-scale and hybrid techniques due to the higher computational cost of 3D processing. CNN-based techniques continue to have drawbacks despite their effectiveness in cardiac image segmentation, such as limited modeling of long-range relationships, large computing costs, and susceptibility to data quality [42].

### 2.2. Attention Modules

To enhance the representation of unique regional traits, medical image segmentation networks have extensively incorporated attention mechanisms in recent years. By adding attention gates to the U-Net framework, NAS-UNet [43] sought to increase the segmentation accuracy of small target structures. Furthermore, segmentation networks are now able to capture more contextual information because of the incorporation of Transformer-based models. To improve feature representations, Woo et al. [44] presented the Convolutional Block Attention Module (CBAM), which integrates channel and spatial attention mechanisms and can be easily incorporated into both classification and segmentation networks. By adding attention gates to the Attention U-Net architecture, Oktay et al. [45] made it possible for the network to concentrate more accurately on ROI.

Transformer architecture [46], which was first created for natural language processing, has lately been modified for medical image segmentation. Transformers offer robust modeling of global dependencies, but they usually need a lot of computing power and lengthy training periods, especially when extracting multiscale features [47,48]. A number of studies [49,50] have investigated Vision Transformer (ViT)-based designs or lightweight attention modules as ways to get around these restrictions. Su et al. [49] proposed SparseViT, for instance, which uses sparse self-attention to lower computing costs while preserving competitive performance on a variety of datasets. To better utilize spatial-spectral information with less computational complexity, Zhang et al. [50] introduced the Transformer-based ESFormer architecture for single-HSI super-resolution, which incorporates spectral-correlation coefficients (SCC) and an effective SCC-core self-attention (ESSA) mechanism.

The Organ-DETR architecture, which combines dense query matching and multiscale attention for 3D organ detection using query-based Transformers, was proposed by Ghahremani et al. [6]. However, its direct applicability to pixel-level segmentation tasks is limited by its detection-oriented design. To semantically segmenting plant organs in 3D point clouds, Liu et al. [51] created TPointNetPlus by fusing PointNet++ with Transformer modules; however, despite encouraging outcomes, its domain specificity and lack of validation on human anatomical data continue to be limits. The OMT-SAM architecture was proposed by Zhang et al. [52] and combines multiscale feature fusion for multi-organ segmentation with CLIP-based image–text prompts. Despite its effectiveness, its dependence on big pre-trained models and fast engineering raises model complexity and may restrict generalization. To improve abdominal organ segmentation, Jha et al. [28] presented MDNet, a multi-decoder architecture that incorporates spatial refinement and multi-scale enhancement modules, albeit at the expense of greater computational complexity. Finally, Shah et al. [44] provided a thorough analysis of 88 Transformer-based segmentation models, highlighting the advantages of U-Net–Transformer hybrids in contextual modeling while simultaneously pointing out the necessity of pretraining and transfer learning in situations with a shortage of labeled data.

When combined, these studies demonstrate the effectiveness of Transformer-based methods for simulating long-range relationships, but they also point out real-world drawbacks in terms of generalization, data requirements, and model complexity.

## 3. Proposed Segmentation Framework

### 3.1. Overall Pipeline Overview

**Figure 1:**
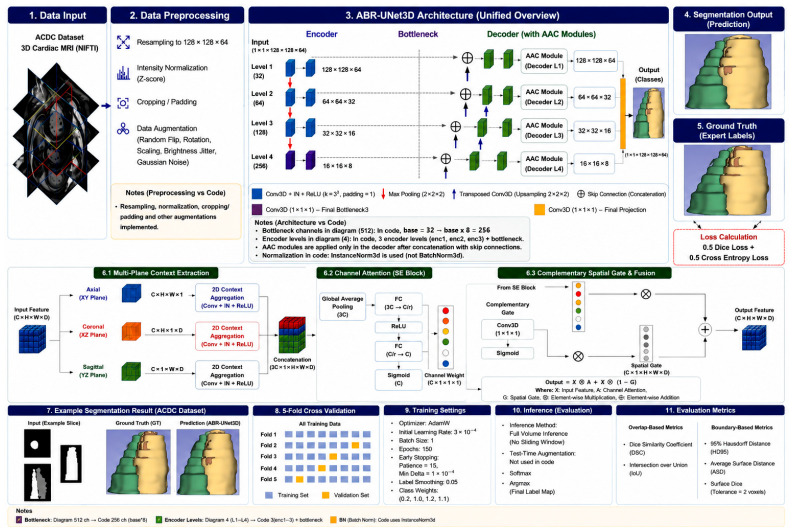
Flowchart of the proposed model.

An end-to-end 3D segmentation pipeline for ACDC cardiac MRI volumes is the design of the proposed method. Figure 1 illustrates the complete ABR-UNet3D segmentation framework. The pipeline consists of data preprocessing, hierarchical 3D encoding, global bottleneck aggregation, AAC-enhanced decoder refinement, hybrid optimization, and multi-metric evaluation. The proposed ABR-UNet3D framework consists of a robust and systematically designed six-stage pipeline: (i) volumetric preprocessing of ACDC MRI images, (ii) hierarchical 3D feature extraction via an encoder, (iii) global context collection at the bottleneck, (iv) boundary-sensitive feature enhancement via AAC-enhanced decoder stages, (v) hybrid Dice–Cross-Entropy-based optimization, and (vi) multi-metric evaluation and reporting. Unlike classical UNet3D, this proposed design incorporates a unique approach, such as optimizing skip-connected decoder features using direction-oriented complementary attention to improve anatomical consistency and boundary determination prior to final classification.

This study addresses a voxel-level multi-class semantic segmentation problem on 3D cardiac MRI volumes. Each voxel is assigned to one of four classes: BG, RV, MYO, and LV. The model aims to estimate class probabilities for each voxel given the input volume and to obtain the final segmentation map using the argmax operation. In this framework, the network produces a logit for each class before the softmax layer; these logits are converted into probabilities, which are then used to compute the loss functions. Class weighting is used to reduce class imbalance because of the multi-class setting, and dice-based loss components are included to enhance shape and boundary sensitivity. The network architecture, training plan, and evaluation processes are set up in accordance with the implementation’s fixed class count of C = 4.

Input/output representation:

X ∈ R ^1×D×H×W^ (1)

(2) $$Z=f_{\theta }(X)\in R^{C\times D\times H\times W}$$

(3) $$P=\mathrm{Softmax}(Z),\;P_{c}\left(v\right)=\frac{e^{Z_{c}(v)}}{\sum_{k=1}^{C}e^{Z_{k}}(v)}$$

Ŷ(v) = argc ∈ {1…C}maxPc(v)(4)

In this case, D, H, and W stand for the volume’s depth, height, and width, respectively, while X is the single-channel 3D MRI input volume. The proposed ABR-UNet3D-based segmentation network with learnable parameters is represented by the function $f_{\theta }(\cdot )$. The network output Z, which has dimensions C × D × H × W, is the logit tensor produced prior to the softmax operation for every class. The softmax function is used to transform these logits into probabilities, producing voxel-level class probability maps represented by P. The class with the highest predicted probability is then chosen for each voxel v, resulting in the final segmentation label volume, which is represented by Ŷ.

### 3.2. Problem Definition and Presentation

#### 3.2.1. Image/Label Pairing

The NIfTI (.nii.gz) format is used to store the dataset’s pictures and masks. The function looks over the image list during preprocessing and looks for a matching ground-truth label file with the same base filename for every image. Due to the possibility of training with wrong labels or tainted evaluation results, this stage is crucial. Images without corresponding labels are skipped with a warning, and if no valid pairs are found in the training or test sets, the process is terminated using an assertion check. In this way, data consistency is ensured from the outset, preventing silent errors from propagating during training. In practice, this approach facilitates early detection of filename mismatches, particularly those arising from manual copying or reorganization of the dataset.

#### 3.2.2. Resize3D

Since the dimensions of the volumes in the ACDC dataset can vary between samples, all volumes were resampled to a common target resolution (128 × 128 × 64) to obtain a constant input size. This process allows the network to work with consistent input tensors. Linear interpolation was used to maintain density continuity for image volumes, and nearest neighbor interpolation was used to preserve class labels for segmentation masks. The rescaling process was performed with scaling coefficients calculated separately for each axis, and the corresponding expression is given in Equation (5). The input image and label maps are transformed using the processes given in Equations (6) and (7), respectively.

(5) $$s=\left(\frac{D_{t}}{D},\frac{H_{t}}{H},\;\frac{W_{t}}{W}\right)$$

X′ = Resize3D(X;s,order = 1)(6)

Y′ = Resize3D(Y;s,order = 0)(7)

Here, X and Y denote the original 3D image volume and the corresponding ground-truth segmentation volume, respectively. The target volume dimensions are set to 128 × 128 × 64, while D, H, and W stand for the original volume’s depth, height, and width. The scale (zoom) factors calculated for each spatial axis are indicated by the variable s. The interpolation technique is determined by the order parameter: order = 0 means nearest-neighbor interpolation for segmentation masks, which guarantees the preservation of discrete label values and label integrity, while order = 1 means linear interpolation for image volumes, which maintains intensity continuity.

#### 3.2.3. Z-Score Normalization

Since the density values in MRI images can vary depending on the device used and the imaging conditions, instance-wise normalization was applied in this process so that the model could focus on relative contrast information instead of absolute density values. Accordingly, each volume is transformed with Z-score normalization with a mean of 0 and a standard deviation of 1. A small ε constant was added to the calculations to ensure numerical stability in low variance volumes. After normalization, clipping was applied to reduce the effect of outliers and obtain a more balanced density distribution. The mathematical expression of Z-score normalization is given in Equation (8):

(8) $$\hat{X}=(X-\mu )/(\sigma +\varepsilon )$$

In this case, the unnormalized 3D MRI input volume is indicated by X. The mean and standard deviation of the voxel intensities inside the volume are denoted by μ and σ, respectively. In this work, ε, a tiny constant included for numerical stability, is set to 10^−8^. The normalized volume produced after z-score normalization is indicated by $\hat{X}$.

#### 3.2.4. Intensity Clipping 

Some voxels may still show high absolute values after z-score normalization (for example, because of noise, metal artifacts, or too bright areas). Because they skew the optimization process toward outlier samples, such extreme values might have a negative impact on model training. To counteract this, normalized intensities are clipped in the implementation to the range [−3, 3]. Additionally, clipping helps avoid numerical instability during training because excessive values can cause activation dynamics to be disrupted, especially when ReLU and Instance Normalization are employed simultaneously. The [−3, 3] interval is frequently used in practice and, assuming a roughly typical normal distribution, keeps most values.

(9) $$\hat{X}=\mathrm{clip}(\hat{X},\;-3,\;3)$$

In this case, $\hat{X}$ stands for the 3D input volume following z-score normalization, and $\hat{X}$ for the final input volume following the clipping process. All voxel intensities outside of this range are set to the appropriate threshold values, while the lower and upper boundaries are set to −3 and 3, respectively.

#### 3.2.5. Data Augmentation (3D Augmentation)

To reduce overlearning during the training process and increase the model’s robustness against noise, contrast, and orientation changes, 3D data enhancement techniques were applied. Data enhancement was performed only during the training phase; no intervention was made to the validation and test data. In the proposed method, the probability of application for each transformation was set to AUG_PROB = 0.6. Within the scope of spatial transformations, volumes were randomly rotated along three axes and subjected to small-angle rotation (between −10° and +10°). The expressions for these processes are presented in Equations (10) and (11). Additionally, density-based transformations were performed to model contrast and brightness changes; this included the addition of Gaussian noise through random scaling and shifting operations. The mathematical expressions for these processes are presented in Equations (12) and (13). To maintain label integrity in segmentation masks, the same transformations were applied using nearest neighbor interpolation (order = 0). This increased data diversity while preventing distortion in class labels.

Flip operation:

(10) $$X_{flip}=\mathrm{Flip}(\hat{X},\;\mathrm{axis}=a)$$

Rotation process:

(11) $$X_{rot}=\mathrm{Rotate}(\hat{X},\alpha )$$

Intensity jitter (scaling and offset):

(12) $$X_{jit}=s\cdot \hat{X}+b$$

Adding Gaussian noise:

(13) $$X_{noise}=\hat{X}+N\;(0,\;{\sigma_{n}}^{2})$$

Here, $\hat{X}$ denotes the preprocessed input volume after normalization and clipping. The variable a represents the spatial axis along which the flip operation is applied, where a ∈ {0, 1, 2}. The parameter α denotes the rotation angle, sampled from the range [−10°, +10°]. The parameter s is a randomly selected intensity scaling factor, chosen from the interval [0.9, 1.1], and b denotes a random intensity offset (brightness shift) selected from [−0.1, 0.1]. Finally, σ represents the standard deviation of the Gaussian noise added to the volume, sampled from the range [0, 0.05]. All these parameters are drawn randomly for each augmentation step.

#### 3.2.6. Data Splitting

The existing training data was randomly divided into two parts, 80% training and 20% validation, and a fixed randomness setting was preferred to ensure reproducibility. The test set was obtained from separate folders to allow for an unbiased evaluation of the model’s generalization performance. A small batch size was preferred during the training process due to the high memory requirements of 3D volume data. In this study, all methods were evaluated using a five-fold cross-validation protocol to ensure a robust and fair comparison. In addition, single-run experiments are reported to provide a direct comparison of best-epoch performance across models. For training and validation, the training data was divided into 80% and 20% sections, respectively. Test performance was assessed using the ACDC test set, which is kept in a directory that is entirely different. The test set was only utilized for final performance evaluation and reporting; it was not used during any training phase.

### 3.3. Encoder–Bottleneck Structure

#### 3.3.1. ConvBlock

The core element of the proposed ABR-UNet3D architecture is a 3D convblock consisting of two consecutive 3D conv layers, each followed by Instance Normalization and ReLU activation. The kernel size in each of the implementation’s 3D convolution layers is set at 3 × 3 × 3 with a padding value of 1. This decision ensures that only MaxPool operations cause changes in spatial resolution while maintaining spatial dimensions during convolution. This prevents size mismatches in the encoder–decoder structure’s skip connections. The following is the mathematical expression for a single 3D convolution layer:

(14) $$F^{\left(l\right)}=\varphi (\mathrm{IN}(W^{\left(l\right)}*F^{(l-1)}+b^{\left(l\right)}))$$

In this case, the input feature map from the previous layer is represented by F^(l−1)^, and the output feature map of the l-th layer is represented by F^(l)^. The learnable weight and bias parameters of the l-th convolution layer are denoted by W^(l)^ and b^(l)^, respectively. The 3D convolution operation is shown by the “∗” operator. The ReLU activation function is shown by φ(.), whereas the Instance Normalization function is represented by IN(.). When these elements are used together, the network’s capacity for generalization is increased and numerical stability is improved.

#### 3.3.2. Encoder

The encoder structure, which makes up the ABR-UNet3D architecture’s downsampling pipeline, is explained here. The encoder gradually extracts multi-scale feature representations by increasing the number of channels, beginning with the input volume. The encoder creates feature maps with 32, 64, and 128 channels at progressively higher levels when the base number of channels is set to base = 32 in the implementation. Learning more complex and context-aware representations is made possible by this growing channel capacity as the spatial resolution drops.

A MaxPool3d layer is used to cut the spatial resolution in half at the conclusion of each encoder stage. Deeper layers can gather more comprehensive contextual information thanks to this downsampling technique. Through skip connections, the intermediate feature maps produced at the various encoder stages (e_1_, e_2_, and e_3_) are sent to the decoder. Fine spatial features that could otherwise be lost during downsampling are recovered by the decoder thanks to these linkages.

In cardiac MRI segmentation, where tissue boundaries may be hazy and contrast levels may be low, this approach is very crucial. A more stable learning process is achieved by applying instance normalization throughout the encoder, which reduces variability in intensity distributions brought on by variations in patients and imaging techniques. Overall, this encoder structure adheres to the traditional 3D U-Net architecture, however the developed AAC module improves the decoder side even more. Equation (15) provides the mathematical expression for the encoder’s downsampling process.

(15) $$F_{\mathrm{pool}}=\mathrm{MaxPool}(F;\;k=2,\;s=2)$$

In this case, F stands for the input feature map and F_pool_ for the output feature map that has been downsampled. In this study, the parameters k and s stand for the pooling kernel size and stride, respectively, and are both set to 2.

#### 3.3.3. Bottleneck

The deepest level between the encoder and decoder is referred to as the bottleneck layer in this context. The network can now aggregate the global contextual information that the encoder collected because the channel capacity is at its maximum and the spatial resolution is at its lowest. In this study, 256 channels—double the number of channels at the highest encoder level—are used to mimic the bottleneck.

In this study, a fixed number of 256 channels in the bottleneck layer was chosen to provide a balanced structure between model capacity and computational cost. Increasing the number of channels, especially in 3D medical imaging tasks, can improve the model’s representational power, but it can also increase the risk of overfitting due to the increased number of parameters. Therefore, an appropriate number of channels was selected to maintain sufficient feature representation capacity without degrading the model’s generalization performance.

Approaches like nnU-Net, which can adapt dynamic channel structures according to data features, offer more flexible solutions. However, a fixed channel structure was preferred in this study to ensure comparability and control of the model design. The effect of adaptively determining the number of channels on model performance can be examined in detail in future studies.

The bottleneck in the proposed model is a single 3D convolutional block (ConvBlock), which consists of two successive 3D convolution layers, each of which is followed by ReLU activation and Instance Normalization. Strong representational ability is necessary for finer structures like the myocardium, and this structure captures both the global geometry of the ventricular chambers and the contextual information around them.

The decoder output may show topological irregularities and blurred boundaries if the bottleneck capacity is inadequate. Thus, in 3D segmentation networks, the number of channels in the bottleneck is a crucial design decision. This study adheres to the widely accepted approach of setting the bottleneck width to twice the depth of the encoder level.

### 3.4. AAC-Enhanced Decoder

#### 3.4.1. Decoder and Skip Connections

The decoder structure and skip links between the encoder and decoder, which together make up the ABR-UNet3D architecture’s upsampling pipeline, are covered in this section. Using ConvTranspose3d layers, the decoder gradually restores spatial resolution. At each stage, it uses skip connections to concatenate the upsampled features with the appropriate encoder features. This method preserves fine spatial features from previous encoder levels while incorporating high-level semantic information from deeper layers.

Three levels make up the decoder in the implementation: up0 → dec0, up1 → dec1, and up2 → dec2. At each level, a ConvTranspose3d layer is used to boost the spatial resolution, and then the matching encoder feature maps are concatenated channel-wise. The number of channels is momentarily doubled by this concatenation, however a ConvBlock is then used to compress and enhance the combined features. This procedure lessens interference from inter-class features while promoting more precise boundary delineation. A 1 × 1 × 1 convolution layer creates logit for each of the four segmentation classes at the last decoder level. The softmax function transforms these logits into voxel-wise class probabilities, and the argmax operation yields the final segmentation volume (BG, RV, MYO, LV). This layer produces class-specific pre-softmax scores by functioning as a voxel-wise linear classifier. The following is a mathematical expression for the channel-wise concatenation procedure utilized in the skip connections between the encoder and decoder:

(16) $$F_{cat}=Concat(F_{up},F_{skip})$$

Here, E_s_ stands for the skip feature map from the associated encoder level that has finer spatial features, and D_u_ for the decoder feature map that was acquired following the upsampling procedure. These two feature maps are concatenated channel-wise by the concat(·) operator.

#### 3.4.2. Aspect-Aware Pooling

In this study, following skip connection fusion, the AAC module extracts anatomical context from multiple spatial perspectives. Since 3D heart structures can exhibit different features along the *x*, *y*, and *z* axes, three anatomical planes—XY (by narrowing the *z*-axis), XZ (by narrowing the *y*-axis), and YZ (by narrowing the *x*-axis)—were used in the design to capture orientation-specific global context. This design is implemented using AdaptiveAvgPool3d with the aim of reducing one spatial dimension at a time and creating three plane-specific channel descriptors. Each descriptor summarizes global contextual information from a different anatomical perspective. The resulting planar summaries are then used for attention generation.

#### 3.4.3. Channel Attention and Complementary Gating

In this process, the three planar descriptors are combined along the channel dimension and passed through a light multilayer perceptron (MLP) and then a sigmoid activation to create a channel attention vector in the range [0, 1]. The resulting vector has the ability to highlight informative channels derived from multiplanar context by multiplying the combined decoder features. In addition, a complementary spatial gating branch is introduced using 1 × 1 × 1 convolution followed by sigmoid activation. The final AAC output is obtained through a residual-style combination of features enhanced by the attention mechanism and gated features. Thanks to this dual mechanism, it enhances class differentiation and improves boundary determination, especially in anatomically challenging regions such as the myocardium. The attention-generating block in this design receives three planar summaries after being combined along the channel dimension. Against typical problems such as axial anisotropy and inter-patient anatomical heterogeneity, this design helps to provide more balanced and selective channel weighting and is particularly suitable for 3D data.

Commonly used attention mechanisms in the literature, such as SE and CBAM, primarily focus on channel-based or channel–spatial reweighting. In contrast, the AAC module proposed in this study not only rescales feature representations but also improves boundary sensitivity by incorporating multi-planar contextual information from the XY, XZ, and YZ planes together with a complementary gating mechanism. This design enables a form of feature refinement beyond conventional attention operations and contributes to better preservation of structural consistency, particularly in anatomically challenging regions such as the right ventricle (RV) and myocardium (MYO). The feature map entering the AAC module can be defined according to Equation (17):

X∈ℝ^{B × C × D × H × W}^ (17)

In the proposed approach, the summaries computed for the three anatomical aspects are formulated as follows: For the XY plane (with the Z-axis compressed):

(18) $$U_{xy}=AvgPool_{xy}(X)\in R^{B\times C\times D\times H\times 1}$$

(19) $$g_{xy}=(1/(D\cdot H)){\;\sum }_{d=1}^{D}\sum_{h=1}^{H}U_{x}y(:,:,d,h,1)\in R^{B\times C}$$

For the XZ plane (with the Y-axis compressed):

(20) $$U_{xz}=AvgPool_{xz}\;(X)\in R^{B\times C\times D\times 1\times W}$$

(21) $$g_{xz}=(1/(D\cdot W))\sum_{d=1}^{D}\sum_{w=1}^{W}U_{x}z(:,:,d,1,w)\in R^{B\times C}$$

For the YZ plane (with the X-axis compressed):

(22) $$U_{yz}=AvgPool_{yz}(X)\in R^{B\times C\times 1\times H\times W}$$

(23) $$g_{yz}=(1/(H\cdot W))\sum_{h=1}^{H}\sum_{w=1}^{W}U_{yz}(:,:,1,h,w)\in R^{B\times C}$$

Finally, the three planar summary channel dimensions are combined to obtain a single vector:

(24) $$g=[g_{xy};g_{xz};g_{yz}]\in R^{B\times 3C}$$

Here, X denotes the feature map entering the AAC module after skip connection fusion at the decoder stage. B represents the batch size (set to 1 in this study), C the number of channels at the corresponding level, and D, H, and W the spatial dimensions of the 3D feature map. The operations AvgPool_xy_, AvgPool_xz_, and AvgPool_yz_ denote adaptive average pooling applied along the Z, Y, and X axes, respectively, reducing the corresponding dimension to one while preserving the other two. These operations summarize global contextual information from the respective anatomical planes. The notation [g_xy_;g_xz_;g_yz_] indicates channel-wise concatenation of the pooled feature descriptors. Table 1 summarizes the layer-wise architectural configuration and parameter distribution of the proposed ABR-UNet3D model.

#### 3.4.4. Channel Attention

Here, in the second stage of the AAC module, the three planar global feature descriptors are transformed into a learnable representation. The aim is to convert them into channel-wise weights. The underlying assumption is that each channel may encode different degrees of discriminative information across the XY, XZ, and YZ planes. Therefore, jointly evaluating g_x__y_, g_xz_, and g_yz_ allows the model to determine the relative importance of each channel, indicating to what extent each channel carries distinctive information. This enables a more accurate assessment of which feature channels should be emphasized.

This transformation is implemented using a two-layer fully connected network (MLP). The first fully connected layer reduces the input dimensionality from 3C to C/r through channel-wise dimensionality reduction. This reduction not only lowers the computational cost but also adds a regularization effect that helps keep the model from giving each channel an unstable or too particular weight. The reduction ratio in this investigation is set at r = 16. The reduced representation is then expanded back to C by the second fully linked layer, giving each channel a unique weight.

Between the two fully connected layers, a ReLU activation function is used to improve representational capacity and add nonlinearity. At the output, a sigmoid activation function constrains the weights to the range [0, 1], resulting in a soft gating mechanism. The resulting channel-attention vector is reshaped to (B, C, 1, 1, 1) and broadcast across the spatial dimensions to perform channel-wise scaling of the feature map. This mechanism enables the decoder to learn which channel combinations, after skip fusion, carry more discriminative information. The computation of the channel-attention vector is formulated as follows:

Combined hash vector:

g ∈ ℝ^{B × 3C}^ (25)

Two-layered MLP structure:

(26) $$h=\delta (W_{1}\;g+b_{1})\in R^{B\times (C/r)}$$

(27) $$a=\sigma (W_{2}\;h+b_{2})\in R^{B\times C}$$

Attention tensor:

(28) $$A=\mathrm{reshape}(a)\in R^{B\times C\times 1\times 1\times 1}$$

Here, W_1_ and b_1_ denote the weight and bias parameters of the first fully connected layer, while W_2_ and b_2_ correspond to those of the second fully connected layer. The parameter r represents the reduction ratio and is set to 16 in this study. The function δ(·) denotes the ReLU activation, and σ(·) denotes the sigmoid activation. The vector a contains the learned channel-attention weights, and the tensor A represents the spatially expanded version of these weights after broadcasting.

#### 3.4.5. Complementary Gate

Here, in the third and final stage of the AAC module, a complementary gating pathway is introduced in addition to the channel-attention mechanism. This solution tackles spatial instabilities that frequently occur in unclear regions of 3D medical images, including cardiac borders, instead of depending just on channel-wise scaling. Although it establishes which channels are more informative, the channel-attention tensor A does not specifically take spatially localized uncertainty into consideration.

A second pathway is established to overcome this restriction. Following skip fusion, the decoder feature map X is run through a 1 × 1 × 1 convolution. A sigmoid function then activates the output to create a spatial gating mask G with values between 0 and 1. This branch, referred to as the complementary gate in the implementation, acts as a spatially aware gating mechanism that can compensate for information that may be overly suppressed by channel attention.

Two complementary scaling routes are used to produce the final AAC output. Whereas the second way uses G for spatial gating, producing X ⊙ G, the first path uses A for channel-wise attention, producing X ⊙ A. The final output is the sum of these two elements, creating a residual-like effect wherein two soft gating mechanisms enhance informative traits instead of strictly filtering them.

More robust feature representations are produced by the AAC module’s ability to treat fused decoder features more judiciously prior to their entry into later convolutional blocks thanks to its complementing design. It is especially helpful for areas with weak borders or poor contrast, as well as for small or fragile anatomical features. From the standpoint of modeling, this complementary mechanism improves the network’s capacity to maintain strong channel-level discrimination while preserving tiny spatial cues. The following is a mathematical definition of the complementary gate and the final AAC output:

Complementary gate:

(29) $$G=\sigma ({\mathrm{Conv}}_{1\times 1\times 1}\left(X)\right)\in R^{B\times C\times D\times H\times W}$$

Aspect-Aware channel scaling:

(30) $$X_{\mathrm{att}}=X⊙A$$

Complementary scaling:

(31) $$X_{\mathrm{comp}}=X⊙G$$

Final AAC output:

(32) $$Y=X_{att}+X_{comp}=X⊙A+X⊙G$$

Here, X denotes the feature map entering the AAC module after skip fusion at the decoder stage. $Conv_{1\times 1\times 1}$ (·) refers to the 1 × 1 × 1 convolution operation, which performs channel mixing and light reparameterization. G represents the complementary gate mask obtained after the sigmoid activation. A denotes the channel-attention tensor with dimensions (B, C, 1, 1, 1), which is broadcast to match the spatial dimensions of X. The operator ⊙ represents element-wise multiplication (Hadamard product). Finally, Y denotes the output of the AAC module, which is passed to the next convolutional block in the decoder.

### 3.5. Training Strategy

#### 3.5.1. Loss Function

This study employs a hybrid loss function combining Soft Dice Loss and Weighted Cross-Entropy with label correction to effectively address class imbalance and boundary sensitivity in multi-class 3D cardiac segmentation. Dice Loss directly optimizes overlap at the region level, Weighted Cross-Entropy improves class separation at the voxel level, and label correction enhances generalization capability by preventing overconfident predictions. The formulation and expression of each component are detailed in the subsections below.

##### Soft Dice Loss

Here, the Soft Dice Loss used for the multi-class 3D cardiac segmentation task is defined. During training, the network outputs are first converted into probability maps using the softmax function. The ground-truth label volume is then converted into one-hot encoding, and the soft Dice coefficient is computed separately for each class. The final Dice loss is obtained by averaging these class-wise Dice losses. Dice-based loss functions directly optimize the overlap between predicted segmentations and ground-truth labels, making them particularly effective for accurate boundary modeling. They are also well suited to medical imaging tasks, where the background class often dominates, and class imbalance is common. Despite having a mini-batch size of 1, the Soft Dice Loss in this study is stable since it is formulated at the voxel level.

To prevent numerical instability when the denominator approaches zero, a small smoothing constant ε is added. This improves the stability of Dice computation, especially for classes with small volumes. Unlike cross-entropy loss, which focuses primarily on voxel-wise class discrimination, the Dice loss emphasizes topological consistency and region-level overlap, helping to address structural properties that cross-entropy alone may fail to capture. The Soft Dice Loss formulation is given by the following equations:

Obtaining class probabilities:

P = Softmax(Z)(33)

A one-hot demonstration of the reality of the earth:

(34) $$Y_{onehot}=OneHot(Y)$$

Soft Dice coefficient for each class:

(35) $$Dice_{c}=(2\sum_{v}P_{c}(v)\cdot Y_{c}(v)+\varepsilon )/(\sum_{v}P_{c}(v)+\sum_{v}Y_{c}(v)+\varepsilon )$$

Multi-class Soft Dice Loss:

(36) $$L_{dice}=1-(1/C)\sum_{c=1}^{C}Dice_{c}$$

Here, $P_{c}(v)$ denotes the predicted probability of class c at voxel v, and $Y_{c}(v$) denotes the corresponding ground-truth value in one-hot encoded form (0 or 1). The summations are taken over all voxels. The constant ε is a smoothing term added for numerical stability and is set to 10^−6^ in this study. C represents the number of classes, which is C = 4 in this work. Finally, $L_{dice}$ denotes the multi-class Soft Dice Loss value.

##### Weighted Cross-Entropy with Label Smoothing

Cross-entropy loss maximizes the predicted probability of the correct class at each voxel. However, class imbalance is common in segmentation tasks (e.g., a large number of background voxels and relatively few myocardium voxels). To address this, class weights are introduced in our approach, defined as [0.2, 1.0, 1.2, 1.1] for the respective classes. This weighting scheme increases the penalty for misclassifying underrepresented classes. In addition, label smoothing is applied to reduce overconfident predictions. Instead of using hard 0/1 targets, the ground-truth distribution is slightly smoothed, which can improve generalization. Together, class weighting and label smoothing help mitigate the effects of class imbalance and reduce the risk of overfitting.

The standard cross-entropy loss is formulated as follows:

(37) $$L_{ce}=-\sum_{v}\sum_{c=1}^{C}w_{c}Y_{c}\;\left(v\right)\log P_{c}\;\left(v\right)$$

The goal of label smoothing is:

(38) $$Y_{c}\left(v\right)=\left(1-\alpha \right)Y_{c}\left(v\right)+\frac{\alpha }{C}$$

(39) $$L_{ce}^{smooth}=-\sum_{v}\sum_{c=1}^{C}\;w_{c}Y_{c}(v)\log P_{c}(v)$$

Here, wc stands for class weight, which in the implementation is set to BG = 0.2, RV = 1.0, MYO = 1.2, and LV = 1.1. C stands for the number of classes (C = 4), log for the natural logarithm, and α for the label smoothing coefficient (set to 0.05).

##### Combined Objective Function

The proposed method combines cross-entropy (CE) loss and dice loss with equal weights. This design balances the overlap and shape sensitivity of Dice loss with the voxel-wise class discrimination capability of CE loss. Using Dice loss alone may lead to suboptimal probability calibration, whereas using CE loss alone may reduce boundary and topological accuracy. In this study, Dice and cross-entropy losses are combined with equal weights (0.5–0.5). Moreover, Dice is also used as the primary validation metric for monitoring model performance during training. The combined loss L is defined as shown in Equation (40).

(40) $$L=0.5L_{dice}+0.5L_{ce}^{smooth}$$

Here, L_dice_ denotes the Dice-based loss and $L_{\mathrm{ce}}^{\mathrm{smooth}}$ denotes the cross-entropy loss with label smoothing. The coefficients 0.5 represent the relative contribution of each component to the total loss.

#### 3.5.2. Optimization and Learning Rate Scheduling

Model parameters are optimized using the AdamW optimizer. Compared to the standard Adam optimizer, AdamW decouples weight decay from the gradient update, leading to more consistent regularization. In the implementation, the initial learning rate and weight decay are defined as fixed hyperparameters. The learning rate is dynamically adjusted during training using the ReduceLROnPlateau scheduler. A factor of 0.5 is applied to the learning rate when the validation loss stops getting better. This approach frequently results in better validation performance and allows for finer updates in later training phases. The scheduler has a five-epoch patience, meaning that the learning rate is decreased if there is no improvement in validation loss for five epochs in a row. Without the need for human learning rate calibration, this method enables the training process to adjust automatically. All experimental studies were conducted on a desktop workstation equipped with an Intel i7-14700F processor, 32 GB of RAM, and an NVIDIA RTX 4070 Super graphics card. The following is a summary of the AdamW optimizer’s parameter updating rule:

(41) $$\theta_{t}=\theta_{\left(t-1\right)}-\eta \cdot ({\hat{m}}_{t}/(\surd {\hat{v}}_{t}+\varepsilon )+\lambda \cdot \theta_{\left(t-1\right)})$$

Here, ${\hat{m}}_{t}$ and ${\hat{v}}_{t}$ stand for the bias-corrected first- and second-order moment estimates utilized in the Adam method, whereas θ_t_ indicates the updated network parameters at step t. The learning rate is represented by η, the weight decay coefficient by λ, and numerical stability is ensured by a tiny constant, ε. When these elements are used together, the optimization process becomes more balanced and the generalization performance is enhanced.

### 3.6. Evaluation Protocol

#### 3.6.1. Metrics

Dice and Intersection over Union (IoU) metrics are used to assess model performance, both as general averages and as per-class comparisons. The overlap between the ground-truth labels and the predicted segmentation is directly measured by these metrics. Each class’s predicted and target masks are binarized in the implementation, and the corresponding intersection and union values are calculated. IoU provides a stricter measure of overlap, whereas Dice is more tolerant to small structures and is widely used in medical image segmentation. In addition to overlap-based metrics, boundary-based evaluation metrics are also employed to provide a more detailed assessment of segmentation quality. Specifically, the 95th percentile Hausdorff Distance (HD95), Average Surface Distance (ASD), and Surface Dice metrics are computed. HD95 captures boundary deviations while being robust to outliers, ASD measures the average boundary distance, and Surface Dice evaluates boundary agreement within a predefined tolerance.

At the end of each epoch, both average and class-wise metric values for training, validation, and test sets are recorded and *stored to ensure reproducibility and facilitate reporting*.

Dice:

(42) $$Dice_{c}=\frac{2\left|P_{c}\cap T_{c}\right|+\epsilon }{\left|P_{c}\right|+\left|T_{c}\right|+\epsilon }$$

where $P_{c}$ denotes the predicted voxel set for class c, $T_{c}$ represents the ground-truth voxel set, and ϵ is a small constant added for numerical stability.

IoU:

(43) $$IoU_{c}=\frac{\left|P_{c}\cap T_{c}\right|+\epsilon }{\left|P_{c}\cup T_{c}\right|+\epsilon }$$

where $P_{c}$ denotes the predicted voxel set for class c, and $T_{c}$ represents the corresponding ground-truth voxel set.

HD95:

(44) $$HD95\left(P,T\right)={\mathrm{percentile}}_{95}\left(max\left(\underset{p\in P}{sup}\underset{t\in T}{inf}d\left(p,t\right),\underset{t\in T}{sup}\underset{p\in P}{inf}d\left(t,p\right)\right)\right)$$

where d(⋅,⋅) denotes the Euclidean distance metric, while P and T represent the predicted and ground-truth surface point sets, respectively.

ASD:

(45) $$ASD\left(P,T\right)=\frac{1}{\left|P\right|+\left|T\right|}\left(\sum_{p\in P}\underset{t\in T}{min}d\left(p,t\right)+\sum_{t\in T}\underset{p\in P}{min}d\left(t,p\right)\right)$$

where d(⋅,⋅) denotes the Euclidean distance metric between surface points.

Surface Dice:

(46) $$SD\left(P,T\right)=\frac{\left|\left\{p\in P:d\left(p,T\right)\leq \tau \right\}\right|+\left|\left\{t\in T:d\left(t,P\right)\leq \tau \right\}\right|}{\left|P\right|+\left|T\right|}$$

where t denotes the tolerance threshold used in Surface Dice computation, which was set to 2 voxels in this study.

#### 3.6.2. Reporting and Visualization

The pipeline in the proposed method produces visual outputs and report files in addition to quantitative measurements to facilitate thorough experimental documentation. Training curves, confusion matrix figures, 3D NIfTI outputs (image, ground truth, and prediction), and 2D cross-sectional visualizations (axial, sagittal, and coronal views) are all automatically saved by the implementation. Additionally, CSV and TXT formats are used to record train, validation, and test metrics for each epoch. Because all intermediate outputs can be stored and reanalyzed, if necessary, this structured reporting approach promotes the transparency of the Experimental Setup and Results sections. Specifically, per-class performance measures and confusion matrices aid in locating mistake patterns unique to a given class. Consequently, the entire pipeline becomes traceable from data processing to final evaluation, and the technique and outcomes stay closely linked.

## 4. Experimental Results

In the experimental analysis, the proposed ABR-UNet3D method was compared with three widely used baseline segmentation approaches: U-Net [53], V-Net [28], and nnU-Net [42]. V-Net was created especially for volumetric (3D) data, nnU-Net is considered a powerful reference framework because of its automatic configuration capabilities, and U-Net is a fundamental encoder–decoder architecture in biomedical segmentation. All models were trained and assessed on the ACDC dataset.

In recent years, transformer-based or pre-trained large-scale models have yielded good results in 3D medical image segmentation. However, such approaches generally require much higher computational costs, large-scale pre-training data, or different training protocols. This makes a direct and fair comparison difficult for the ACDC dataset, which contains limited data and is evaluated under standard training settings. The main objective of this study is to analyze the effect of the proposed AAC module on classical and commonly used 3D segmentation architectures in isolation. For these reasons, comparisons are limited to reference models that can be trained under the same data splitting, the same training strategies, and similar parameter budgets.

In this study, the fully automated self-configuring pipeline commonly used in the literature for nnU-Net was not chosen; instead, all models were retrained under the same data splitting and training settings to ensure consistent comparison among all models. The main purpose of this preferred approach is to more clearly demonstrate that performance differences stem directly from the model architecture rather than from the data processing steps.

To create a fair comparison, same training conditions were applied across all approaches. These comprised the same input volume size (128 × 128 × 64), batch size (1), total number of epochs (130), and starting learning rate (5 × 10^−5^). Additionally, all models utilized the same learning rate scheduler. Furthermore, the test set was maintained in strict isolation and the same training–validation split (80–20%) was used. Every network was trained for the entire 130 epoch; no model was subjected to early halting. Using the same parameters, all comparison models were also subjected to the data augmentation procedures employed in the proposed method: Gaussian noise addition with AUG_PROB = 0.6, intensity scaling and contrast variation, and random axis flips and rotations. This helped ensure that architectural variations, rather than differences in training methods or data diversity, were the primary source of performance differences. Consequently, the assessment procedure was created to be impartial, repeatable, and trustworthy. The ACDC dataset serves as the basis for the performance measures presented in the experimental results. Intersection over Union (IoU) and Dice Similarity Coefficient (DSC) were computed as overall averages as well as for each class (BG, RV, MYO, LV).

In this study, the model selection process was performed solely based on the DSC in the validation set. All models were trained for a fixed 130 epoch without early stopping. In addition, in our experimental studies, the test set was kept completely independent and was not used for model updates, hyperparameter adjustments, or selection decisions at any stage of the training process. Thus, the aim was to ensure an unbiased evaluation of generalization performance.

The training dynamics of the proposed ABR-UNet3D model across 130 epochs on the ACDC dataset are compiled in Table 2. Early in training, the model demonstrated quick performance gains (e.g., achieving a test DSC of 0.8978 by epoch 10), and in subsequent epochs, it steadily stabilized. On the difficult ACDC cardiac MRI dataset, the highest test DSC value of 0.9603 was recorded at epoch 130, indicating significant generalization potential. The test set was kept completely separate from the training and validation procedures, even though test metrics are presented across epochs for transparency and completeness. Validation performance was the sole criterion used to choose the model and its hyperparameter settings. Test metrics were not utilized to affect model optimization or selection; they were solely recorded for post hoc analysis. Consequently, no data from the test set was included in the training process, guaranteeing an objective assessment of generalization performance.

The class-wise test set metrics at epoch 130, where the proposed UNet3D + AAC approach performed at its peak, are shown in Table 3. With very good findings in difficult anatomical classes like the right ventricle (RV: 0.9485 DSC) and myocardium (MYO: 0.9211 DSC), the mean test DSC is 0.9603. These results demonstrate the role of the AAC attention module, particularly for highly changeable and physically tiny tissues such as the RV. Performance on the background (BG) class is extremely high (DSC: 0.9989), as anticipated. The proposed method’s dependability for precise cardiac MRI segmentation is further supported by the IoU values, which exhibit a similar pattern.

The proposed ABR-UNet3D model’s best test set performance is shown in Table 4 when compared to the conventional UNet3D, V-Net, and nnU-Net architectures. The proposed approach outperforms all examined models in terms of overall average metrics, with a mean Dice score (DSC) of 0.9603 and a mean IoU of 0.9261. The improvements are further highlighted by a class-wise analysis, especially in the segmentation of the right ventricle (RV) and myocardium (MYO). The AAC module’s efficacy in teaching boundary-sensitive and resilient feature representations is demonstrated by the higher and more consistent DSC values attained in these classes. These findings imply that the proposed architecture provides more consistent performance in clinically significant cardiac structures in addition to increasing overall segmentation accuracy. This comparison is based on the best performance values obtained under the single training–test split. In this study, a five-fold cross-validation was also performed to evaluate the generalizability of the model against different data splits, and the results obtained are presented in Table 5.

Table 5 presents the results of the five-fold cross-validation performed for the proposed ABR-UNet3D model along with other models (V-Net, nnU-Net, and UNet3D) used for comparison. The findings reveal the performance of all models across different data divisions and allow for a comprehensive evaluation of the generalizability of the proposed method. The results show that the proposed ABR-UNet3D model performs better than other methods in both overlap-based metrics (DSC and IoU) and boundary accuracy metrics (HD95, ASD, and Surface Dice). The significant improvement in HD95 and ASD values indicates that the model can perform more precise and consistent segmentation in boundary regions. The high values obtained in the Surface Dice metric reveal that the segmentation results are quite successful in terms of surface fit.

To more thoroughly evaluate the boundary accuracy of the proposed method, HD95, ASD, and Surface Dice metrics were calculated, and the results are presented in Table 6. The low HD95 and ASD values obtained, particularly in anatomically difficult-to-segment regions such as RV and MYO, indicate that the model can produce stable and accurate predictions in boundary regions. The high Surface Dice values, on the other hand, reveal that the segmentation results are quite successful in terms of surface fit. Distance-based metrics (HD95 and ASD) were calculated in millimeters after resampling all volumes to isotropic resolution during the preprocessing phase. The Surface Dice metric was evaluated with a tolerance of 2 voxels.

Cohen’s d effect size statistical analysis was performed on the experimental results obtained from the proposed and baseline models, and the results are presented in Table 7. In this analysis, mean and standard deviation values were considered for each model. Comparison was made based on mean performance differences and Cohen’s d effect size. Since interpretation based solely on *p*-values is limited in terms of statistical power in a small cross-validation sample such as n = 5, this effect size-based approach provides a more explanatory framework. High values in DSC, IoU, and Surface Dice metrics; and low values in HD95 and ASD metrics indicate better performance.

The Cohen’s d effect size values presented in Table 7 reveal that the proposed ABR-UNet3D model exhibits significantly higher performance compared to the other methods across all metrics. Differences with V-Net and nnU-Net are particularly pronounced in boundary-based metrics (HD95, ASD, and Surface Dice), while the difference against UNet3D is very high across all metrics. The fact that all Cohen’s d values are well above the d ≥ 0.8 large effect size threshold indicates that the observed performance differences are significant not only numerically but also practically.

**Figure 2:**
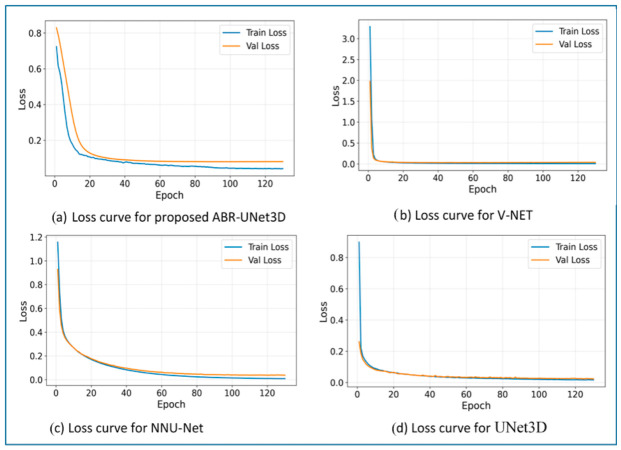
Loss curves for the 4 approaches used in the testing process.

**Figure 3:**
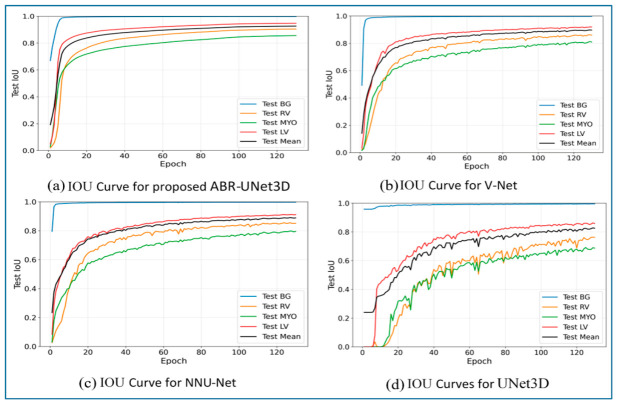
IOU curves of the approaches used in the testing process.

**Figure 4:**
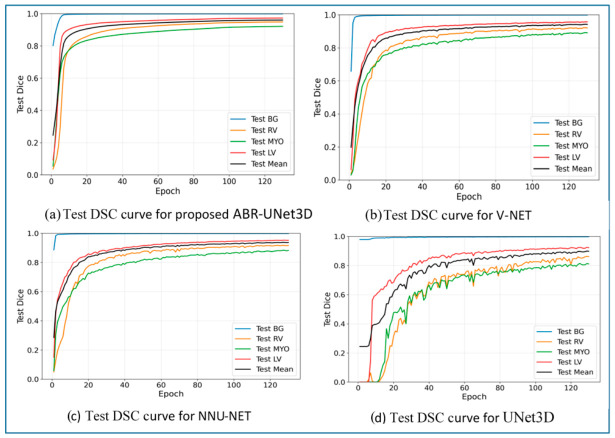
DSC Curves of Approaches Used in the Testing Process.

Figure 2, Figure 3 and Figure 4 present the training curves (loss, IoU, and DSC metrics) of the proposed ABR-UNet3D model. Examination of the curves reveals a rapid decrease in the loss value and a corresponding rapid increase in IoU and DSC metrics in the early stages of the training process. In subsequent epochs, the performance continues more gradually, and the model exhibits stable learning behavior. This indicates the absence of a significant overfitting tendency. These trends observed in Figure 2, Figure 3 and Figure 4 are consistent with the quantitative results presented in Table 2, Table 3, Table 4, Table 5 and Table 6. Furthermore, the cross-validation results presented in Table 5 support the generalizability of the model, showing similar performance across different data sets. In addition, the effect size analysis (Cohen’s d) presented in Table 7 demonstrates that the obtained performance differences are significant not only numerically but also practically. When all these findings are evaluated together, it is seen that the proposed ABR-UNet3D architecture exhibits strong, stable, and consistent performance on the ACDC dataset.

**Figure 5:**
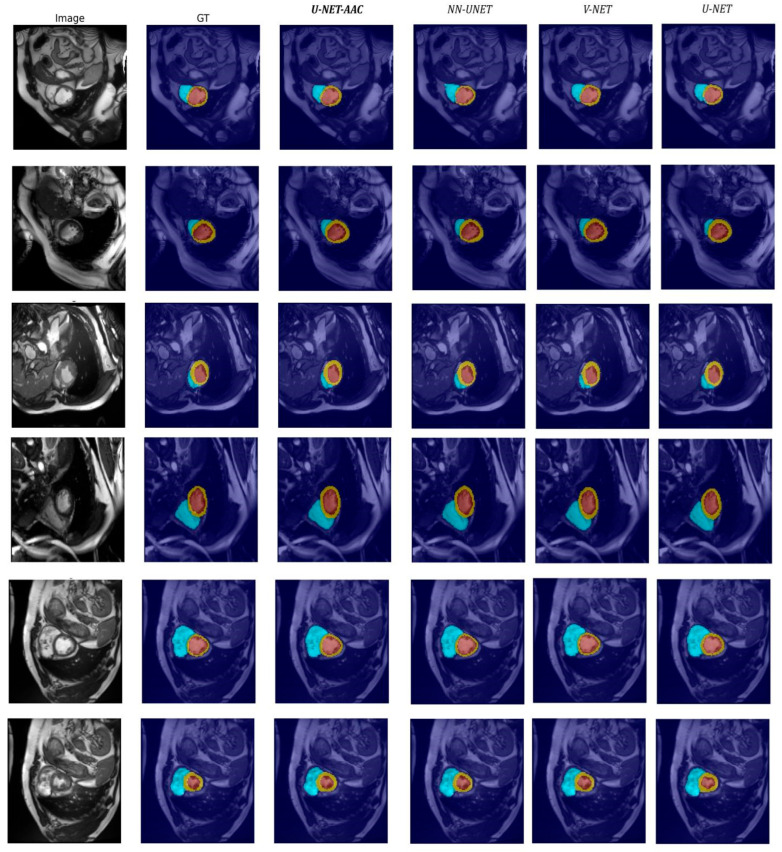
Qualitative comparison of 2D segmentation results obtained from the proposed ABR-UNet3D model and baseline methods on axial cardiac MRI slices selected from different patients in the ACDC dataset. Different colors indicate different cardiac structures/classes (RV, MYO, and LV), while GT represents the ground-truth annotations.

Figure 5 presents a qualitative comparison of 2D segmentation results from axial cardiac MRI slices of six patients selected from the ACDC dataset. The predictions of the proposed ABR-UNet3D model are compared with those of nnU-Net, V-Net, and standard U-Net, alongside the ground truth (GT). Each row corresponds to a different patient. From left to right, the original axial MRI image, the GT label, and the predictions of the four models are shown. Visual inspection indicates that the ABR-UNet3D model achieves the closest anatomical agreement with the GT. In particular, the shape and size of the right ventricle (RV, shown in red) closely match the reference, while the myocardium (MYO, shown in green) exhibits more consistent thickness and continuity. Additionally, the margins of the left ventricle (LV, indicated in orange/yellow) are smoother and more anatomically realistic. The comparison models, on the other hand, exhibit the following typical limitations: standard U-Net performs the worst, with incomplete RV regions and notably erroneous MYO and LV boundaries; V-Net displays more boundary fluctuations and inconsistencies; and nnU-Net tends to underestimate the RV and exhibits local irregularities in the myocardium. These qualitative findings support the AAC module’s contribution, especially in RV segmentation and boundary refining, and are in line with the quantitative findings shown in Table 2, Table 3, Table 4, Table 5 and Table 6. In addition, the cross-validation results presented in Table 5 further confirm the consistency of these observations across different data splits.

**Figure 6:**
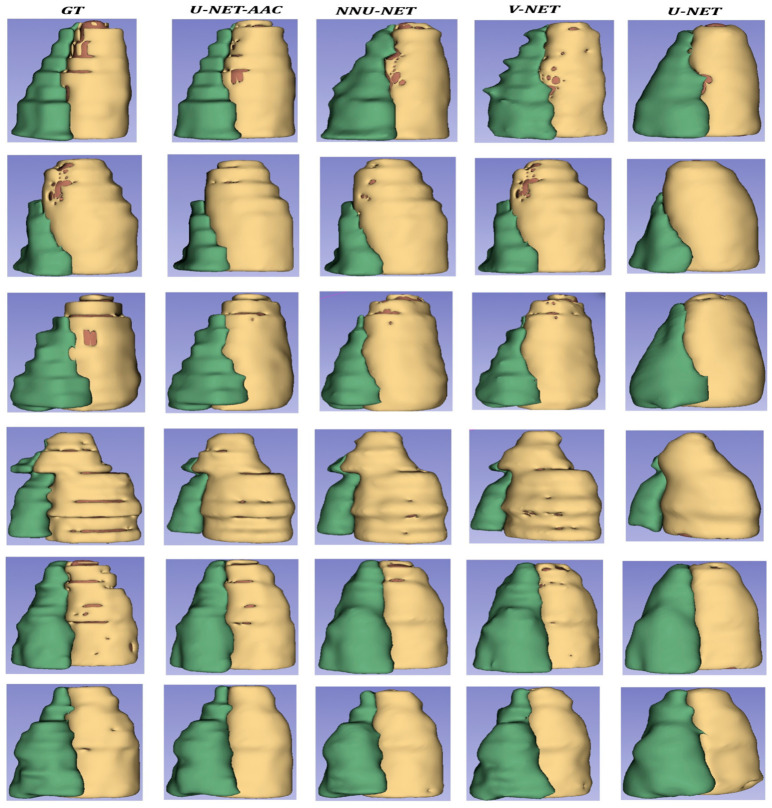
3D segmentation results of the methods used in experimental studies and their corresponding GT images.

The 3D cardiac MRI segmentation results for the same patients are shown in comparison in Figure 6. The GT and the predictions of the four models are shown from left to right, with each row representing a patient. The best visual agreement with the GT volumes is demonstrated by the proposed ABR-UNet3D model. The model offers a more reliable depiction in low-contrast and anatomically complicated regions, as evidenced by the continuity of MYO and LV borders and the preservation of fine RV structural details. The AAC module, which combines Aspect-Aware channel attention with a complementary spatial gating mechanism, is responsible for this visual improvement. By modeling multi-planar (XY, XZ, YZ) contextual information and refining decoder features in a boundary-sensitive manner, the AAC module enhances structural consistency. 

In contrast, nnU-Net and V-Net predictions exhibit more surface irregularities and local topological inconsistencies, particularly in the RV and MYO regions, while the standard U-Net produces coarser segmentations with reduced anatomical continuity. These qualitative findings align with the numerical results reported in Table 2, Table 3, Table 4, Table 5 and Table 6, where the proposed ABR-UNet3D achieved a mean Dice score of 96.03% on the test set, indicating more stable and reliable performance, especially for clinically important and challenging cardiac structures.

To evaluate the computational cost of the proposed ABR-UNet3D model, the number of trainable parameters and inference time were analyzed. Since the number of parameters is a fixed quantity dependent on the model architecture, it was reported as a single value, and the results are presented in Table 8. The model has a total of 5,707,780 trainable parameters. The inference time was measured on the test set using the trained model and was found to be an average of 0.2054 s per volume. In this study, inference time calculations were not included in the cross-validation process. The main reason for this is that inference time depends primarily on the model architecture and the hardware specifications and does not significantly vary across different folds. Therefore, the reported inference time reflects the computational efficiency of the model in practical usage.

An ablation study was conducted to analyze the contribution of different components within the proposed ABR-UNet3D framework, and the results are presented in Table 9. All variants were trained and evaluated under identical conditions, including the same data split, data augmentation strategy, loss function (Cross-Entropy + Soft Dice), optimizer, and learning rate schedule. The ablation results indicate that the baseline UNet3D architecture already provides a strong initial performance. In contrast, the proposed AAC module, integrated at the decoder stage, processes skip-connected features together with multi-planar contextual information and more effectively highlights anatomically relevant regions through its complementary gating mechanism. To better isolate the contribution of each component of the AAC module, an additional component-level analysis is included. This analysis separately evaluates the effects of multi-planar context modeling and complementary gating.

As shown in Table 9, the AAC module introduces a complementary refinement effect that recovers information potentially suppressed during feature propagation, rather than merely rescaling feature responses. This leads to consistent improvements in both Dice and IoU metrics. In addition, class-wise DSC values (RV, MYO, and LV) are included to further analyze the impact of each component on anatomically challenging structures. The results further indicate that while individual components provide moderate gains, the combined use of multi-planar context and complementary gating yields the most significant performance improvement. Improvements are more pronounced in RV and MYO classes, which are known to be more challenging due to anatomical variability and boundary ambiguity. These findings suggest that the performance gain of the ABR-UNet3D model originates from the joint effect of multi-plane contextual modeling and complementary gating mechanisms.

Table 10 presents a comparison of the proposed method with selected studies that also report results on the ACDC dataset, based on Dice Similarity Coefficient (DSC) values. As shown in the table, the proposed approach achieves consistently higher or competitive DSCs in RV, MYO, and LV segmentation compared with the reported results of the referenced methods and also obtains the highest or near-highest average DSC value among the compared approaches. It should be noted that the values reported in Table 10 correspond to single-split evaluation results, which are consistent with the evaluation protocols used in the compared studies. In addition, the robustness of the proposed method has been further validated through five-fold cross-validation, as presented in Table 5.

These findings indicate that the proposed ABR-UNet3D architecture performs competitively on the ACDC dataset and shows competitive effectiveness relative to selected ACDC-based methods in the literature. In particular, the improvements are more pronounced in RV and MYO classes, which are known to be more challenging due to their anatomical variability and boundary ambiguity.

## 5. Discussion

Accurate segmentation of cardiac structures is clinically crucial for reliable calculation of important parameters such as ventricular volumes, ejection fraction, and myocardial mass. Segmentation errors, particularly in areas with high boundary ambiguity, can affect these quantitative measurements and lead to clinically significant deviations. The proposed ABR-UNet3D model’s successful boundary determination process, especially in anatomically challenging segmentation areas such as the myocardium and right ventricle, may support more consistent estimation of these parameters in ACDC-based experimental settings.

In this study, the integration of the proposed AAC module into the decoder phase significantly improved segmentation performance, particularly in structures with high boundary ambiguity such as the RV and MYO. While classical UNet3D architectures mostly rely on local features, the AAC module combines global contextual information from three different anatomical planes, performs channel-level reweighting, and further enhances boundary sensitivity through a complementary spatial gating mechanism. In this study, a sigmoid activation function was chosen for the complementary gate structure in the AAC module. Although the saturation behavior of the sigmoid function is known, no significant gradient-related issues were observed in this study. However, the effect of alternative activation functions such as h-swish on this structure will be explored in future work.

While standard attention mechanisms in the literature generally rely on unidirectional channel or spatial attention, the proposed approach incorporates multi-plane contextual modeling. This enables more consistent performance, particularly in low-contrast and morphologically variable cardiac structures. Clinically, improvements in RV and MYO segmentation may contribute to more reliable estimation of parameters such as ventricular volume and ejection fraction.

The class weights and label smoothing coefficient used in this study were determined to reduce class imbalance and to support more balanced learning of small anatomical structures. However, a systematic sensitivity analysis of these hyperparameters was not conducted within the scope of this study. Investigating the impact of different parameter settings on model performance and generalization is considered a potential direction for future work.

The single-center nature of the ACDC dataset used in this study limits the direct evaluation of the model’s generalization performance across data from different centers and different MRI devices. Although the model’s stability under different data splits has been improved through data augmentation strategies and cross-validation approaches, evaluations on multi-center datasets will allow for a more comprehensive analysis of the model’s generalizability. This will be addressed in future studies.

## 6. Conclusions

This study presents a 3D cardiac MRI segmentation framework enhanced with an Aspect-Aware Complementary Attention (AAC) module. The proposed ABR-UNet3D architecture is designed to produce more selective and robust feature representations, particularly in the myocardium (MYO) and right ventricle (RV), where boundary ambiguity and inflammation-related distortions are common. By combining multi-planar (XY, XZ, YZ) channel-level contextual modeling with a complementary spatial gating mechanism, the AAC module aims to improve boundary sensitivity and structural consistency. Extensive experiments on the ACDC dataset showed that the proposed method achieved a mean Dice score of 96.03% in single-run test evaluations. In addition, five-fold cross-validation experiments yielded an average DSC of 95.2% and IoU of 90.8%, supporting the stability of the proposed framework across different data splits within the evaluated dataset. In comparative evaluations, ABR-UNet3D demonstrated more balanced and consistent performance than standard UNet3D, V-Net, and nnU-Net architectures, especially in the challenging MYO and RV classes. Class-wise evaluations showed that the proposed method reduced inter-class performance variation, improved overall segmentation accuracy, and produced more reliable predictions for clinically significant heart structures. Overall, the results suggest that the proposed architecture provides a stable and competitive segmentation framework for cardiac MRI images, particularly in regions with substantial boundary ambiguity, within the scope of the ACDC dataset. Nevertheless, since the current evaluation is limited to the ACDC dataset, additional validation on larger and more diverse cardiac MRI datasets will be important for further assessing the generalizability of the proposed approach.

## Figures and Tables

**Table 1:** Layer-based architectural details and parameter distribution of the proposed ABR-UNet3D architecture.

Stage	Layer	Operation	Kernel/Stride	Cin → Cout	Output Size (C × D × H × W)	Parameter
Input	—	Input volume	—	1	(1, 128, 128, 64)	0
Encoder-1	enc1-1	Conv3D + IN + ReLU	3 × 3 × 3/1	1 → 32	(32, 128, 128, 64)	896
	enc1-2	Conv3D + IN + ReLU	3 × 3 × 3/1	32 → 32	(32, 128, 128, 64)	27,680
	pool1	MaxPool3D	2 × 2 × 2/2	—	(32, 64, 64, 32)	0
Encoder-2	enc2-1	Conv3D + IN + ReLU	3 × 3 × 3/1	32 → 64	(64, 64, 64, 32)	55,424
	enc2-2	Conv3D + IN + ReLU	3 × 3 × 3/1	64 → 64	(64, 64, 64, 32)	110,720
	pool2	MaxPool3D	2 × 2 × 2/2	—	(64, 32, 32, 16)	0
Encoder-3	enc3-1	Conv3D + IN + ReLU	3 × 3 × 3/1	64 → 128	(128, 32, 32, 16)	221,312
	enc3-2	Conv3D + IN + ReLU	3 × 3 × 3/1	128 → 128	(128, 32, 32, 16)	442,496
	pool3	MaxPool3D	2 × 2 × 2/2	—	(128, 16, 16, 8)	0
Bottleneck	bott-1	Conv3D + IN + ReLU	3 × 3 × 3/1	128 → 256	(256, 16, 16, 8)	884,992
	bott-2	Conv3D + IN + ReLU	3 × 3 × 3/1	256 → 256	(256, 16, 16, 8)	1,769,984
Decoder-2	up2	Transposed Conv3D	2 × 2 × 2/2	256 → 128	(128, 32, 32, 16)	262,272
	concat	Skip (e3)	—	128 + 128	(256, 32, 32, 16)	0
	AAC-2	FC + FC + 1 × 1 × 1 Conv	—	256	(256, 32, 32, 16)	81,920
	Dec2-1	Conv3D + IN + ReLU	3 × 3 × 3/1	256 → 128	(128, 32, 32, 16)	884,864
	Dec2-2	Conv3D + IN + ReLU	3 × 3 × 3/1	128 → 128	(128, 32, 32, 16)	442,496
Decoder-1	up1	Transposed Conv3D	2 × 2 × 2/2	128 → 64	(64, 64, 64, 32)	65,600
	concat	Skip (e2)	—	64 + 64	(128, 64, 64, 32)	0
	AAC-1	FC + FC + 1 × 1 × 1 Conv	—	128	(128, 64, 64, 32)	20,480
	Dec1-1	Conv3D + IN + ReLU	3 × 3 × 3/1	128 → 64	(64, 64, 64, 32)	221,248
	Dec1-2	Conv3D + IN + ReLU	3 × 3 × 3/1	64 → 64	(64, 64, 64, 32)	110,720
Decoder-0	up0	Transposed Conv3D	2 × 2 × 2/2	64 → 32	(32, 128, 128, 64)	16,416
	concat	Skip (e1)	—	32 + 32	(64, 128, 128, 64)	0
	AAC-0	FC + FC + 1 × 1 × 1 Conv	—	64	(64, 128, 128, 64)	5120
	dec0-1	Conv3D + IN + ReLU	3 × 3 × 3/1	64 → 32	(32, 128, 128, 64)	55,392
	dec0-2	Conv3D + IN + ReLU	3 × 3 × 3/1	32 → 32	(32, 128, 128, 64)	27,680
Output	outc	Conv3D	1 × 1 × 1/1	32 → 4	(4, 128, 128, 64)	132
AAC (Total)	—	—	—	—	—	107,520
Model (Total)	—	—	—	—	—	≈5.6 M

**Table 2:** Training Process Metrics of the Proposed Method (Selected Epochs).

Epoch	Loss of Education	Verification DSC	Test DSC	Verification IoU	Test IoU
1	0.7233	0.2521	0.2462	0.1961	0.1914
10	0.1682	0.859	0.8978	0.7693	0.8261
20	0.113	0.8857	0.9271	0.8055	0.8708
30	0.0921	0.8972	0.9359	0.8217	0.886
40	0.0794	0.9043	0.9415	0.8323	0.8952
50	0.0705	0.9095	0.9457	0.8403	0.9028
60	0.0638	0.9134	0.9489	0.8463	0.9083
70	0.0584	0.9165	0.9514	0.851	0.9124
80	0.054	0.919	0.9534	0.8548	0.9156
90	0.0504	0.921	0.9551	0.8579	0.9183
100	0.0474	0.9226	0.9565	0.8604	0.9205
110	0.0449	0.9238	0.9577	0.8624	0.9223
120	0.0428	0.9243	0.9587	0.8638	0.9238
130	0.0412	0.9244	0.9603	0.867	0.9261

**Table 3:** Best Test Results of the Proposed Method (by Class).

Metric Type	Average	BG	RV	MYO	LV
Test DSC	0.9603	0.9989	0.9485	0.9211	0.9726
Test IoU	0.9261	0.9978	0.904	0.8555	0.9472

**Table 4:** Comparison of the Proposed Method with Other Models (Best Test Metrics).

Model	Best Epoch	Mean Test DSC	Mean Test IoU	BG DSC	RV DSC	MYO DSC	LV DSC
Proposed ABR-UNet3D	130	0.9603	0.9261	0.9989	0.9485	0.9211	0.9726
V-Net	129	0.9426	0.8978	0.9986	0.9218	0.8927	0.9573
nnU-Net	127	0.9381	0.8904	0.9985	0.9182	0.8835	0.952
U-Net3D	129	0.8994	0.8276	0.9975	0.8619	0.8135	0.9248

**Table 5:** Comparative Five-Fold Cross-Validation Performance (Proposed) and Baseline Results.

Model	DSC	IoU	HD95 (mm)	ASD (mm)	Surface Dice
Proposed ABR-UNet3D	0.952 ± 0.009	0.908 ± 0.012	1.46 ± 0.21	0.36 ± 0.05	0.988 ± 0.003
V-Net	0.935 ± 0.012	0.881 ± 0.014	2.05 ± 0.34	0.52 ± 0.08	0.972 ± 0.006
nnU-Net	0.930 ± 0.014	0.874 ± 0.016	1.85 ± 0.30	0.48 ± 0.07	0.975 ± 0.005
UNet3D	0.892 ± 0.018	0.813 ± 0.020	2.80 ± 0.45	0.75 ± 0.10	0.955 ± 0.010

**Table 6:** Boundary-Based Evaluation Metrics of Proposed Method.

Class	HD95 (mm)	ASD (mm)	Surface Dice
BG	0.28	0.06	0.997
RV	1.55	0.41	0.981
MYO	1.28	0.31	0.994
LV	1.31	0.28	0.995
Average (FG)	1.38	0.33	0.990

**Table 7:** Cohen’s d effect size values against the proposed ABR-UNet3D model.

Comparison	DSC d	IoU d	HD95 d	ASD d	Surface Dice d	Interpretation
ABR-UNet3D vs. V-Net	1.60	2.07	2.09	2.40	3.37	Large/very large
ABR-UNet3D vs. nnU-Net	1.87	2.40	1.51	1.97	3.15	Large/very large
ABR-UNet3D vs. UNet3D	4.22	5.76	3.82	4.93	4.47	Very large

**Table 8:** Computational Complexity Analysis.

Model	Trainable Parameters	Inference Time (s/volume)
Proposed ABR-UNet3D	5.71 M	0.2054

**Table 9:** Component-wise Ablation Study of the Proposed AAC Module with Class-wise DSC Results.

Model	Channel Attention	Gate	Multi-Planar	Mean DSC	Mean IoU	RV DSC	MYO DSC	LV DSC
UNet3D	No	No	No	0.8994	0.8276	0.8619	0.8135	0.9248
UNet3D + Gate Only	No	Yes	No	0.9120	0.8450	0.8850	0.8350	0.9400
UNet3D + Multi-planar Only	No	No	Yes	0.9320	0.8820	0.9100	0.8600	0.9550
ABR-UNet3D (Full AAC)	Yes	Yes	Yes	0.9601	0.9260	0.9485	0.9211	0.9726

**Table 10:** Comparison table of our proposed approach based on DSC with some studies using the ACDC dataset.

Methods	RV (%)	Myo (%)	LV (%)	Average (%)
TransUNet [54]	88.86	84.54	95.73	89.71
Swin-UNet [45]	88.55	85.62	95.83	90
MISSFormer [55]	86.36	85.75	91.59	87.9
UNETR++ [56]	91.89	90.61	96	92.83
PCCTrans [57]	90.55	90.57	96.22	92.45
DS-UNETR++ [58]	92.23	90.82	96.04	93.03
Our Proposed ABR-UNet3D	94.85	92.11	97.26	94.73

## Data Availability

The data used in this study are publicly available from the ACDC (Automated Cardiac Diagnosis Challenge) dataset [6]. The dataset can be accessed through the official ACDC challenge repository: https://www.creatis.insa-lyon.fr/Challenge/acdc/ (accessed on 12 November 2025). No new data were generated in this study.
